# Sex differences in elite ski mountaineering aerobic performance

**DOI:** 10.3389/fspor.2025.1534315

**Published:** 2025-02-13

**Authors:** Forrest Schorderet, Justin Mottet, Aurélien Lathion, Antoine Raberin, Nicolas Bourdillon, Grégoire P. Millet

**Affiliations:** Institute of Sport Sciences, University of Lausanne, Lausanne, Switzerland

**Keywords:** female athletes, ski mountaineers, maximal oxygen uptake, ventilatory thresholds, Olympic sports, body composition, mountain sports

## Abstract

Ski mountaineering (SkiMo) sprints will debut as an Olympic sport in 2026, yet research on the discipline remains scarce compared to other winter sports. The demanding sprint format, with most of the race time spent on uphill sections, highlights the importance of body composition and maximal oxygen consumption (V˙O_2max_). While previous studies have primarily focused on male athletes, this study aimed to analyze sex differences in physiological parameters of elite SkiMo athletes, hypothesizing that differences in vertical velocities (vV) would surpass those in V˙O_2_ at ventilatory thresholds (VT_1_, VT_2_) and maximal intensity (MAX), respectively. Twenty elite/worldclass Swiss SkiMo athletes (6 women, 14 men, aged 20–32 years) participated in the study. They performed a graded exercise test to exhaustion on a treadmill set at a 25% slope, with breath-by-breath gas exchanges. Elite female SkiMo athletes had a V˙O_2_ value 13.6% lower at MAX (64.0 ± 3.8 vs. 72.8 ± 5.5 ml/kg/min; *p* = 0.002) and 15.5% lower at VT_2_ (54.8 ± 2.8 vs. 62.2 ± 5.8 ml/kg/min; *p* = 0.009) than their male counterparts. Interestingly, the sex-differences in vV at both MAX (1,825 ± 113 vs. 2,125 ± 156 m/h; *p* < 0.001; 16.4%) and VT_2_ (1,412 ± 56 vs. 1,696 ± 151 m/h; *p* < 0.001; 20.1%) intensities were consistently larger than the differences in V˙O_2_. Moreover, fat mass was higher in females (15.2 ± 1.0 vs. 6.6 ± 0.6%; *p* = 0.004). Additionally, vertical running energy cost at VT_2_ was significantly higher in females compared to males (2,329 ± 95 vs. 2,199 ± 60 ml/kg/kmv; *p* = 0.018). Sex differences in uphill velocities (16.4–20.1%) exceeded those in V˙O_2_ (13.6–16.5%). Investigation on the underlying mechanisms is required but several factors may contribute to this pronounced sex difference in uphill velocity beyond aerobic power alone. Overall, the present findings align with recent studies reporting a 16%–20% difference in performance times when investigating sex differences in uphill displacement. The performance gap between men and women appears to be larger in uphill sports.

## Introduction

The 2026 Winter Olympic games are just around the corner, and for the first time since 1924, ski mountaineering (SkiMo) will be featured as an Olympic discipline (1). SkiMo has gained considerable popularity (2) and the inclusion of this sport in the Olympics is likely to further boost its interest (3). However, relatively little research has been conducted on SkiMo compared to other winter endurance sports like cross-country skiing (2). A recent review on sex differences in Olympic winter endurance sport highlighted that scientific research on SkiMo was scarce (4). SkiMo involves a combination of aerobic endurance, muscular strength, and technical proficiency, reflecting the diverse physiological and technical demands of the sport (5). The sprint format, which will be featured in Cortina 2026, is highly specific and consists of a series of 3–4 short, intense rounds of exercise lasting 3–3.5 min each, with rest periods of approximately 20–30 min in between. During each round, skiers complete an uphill section consisting of three successive segments: “ski-run-ski”. During the transitions, they need to manipulate their bindings, skis, and skins. The round concludes with a short downhill ski slalom segment (6). A recent study revealed that uphill performance constitutes 80%–89% of the total race time for males and 80%–93% for females, making it the primary factor distinguishing top sprint ski mountaineers at the international level (7). During the climb, as for any uphill displacement, percent body fat (%BF) is speculated to have a significant impact on performance (7, 8). A recent study reported a significant correlation between “vertical” SkiMo race times %BF (*r* = −0.67; *p* = 0.025) (9) explaining the request of a relatively low body fat mass and light equipment to improve climbing efficiency (5). Two other key factors for SkiMo performance seem to be the maximal oxygen consumption (V˙O_2max_) and V˙O_2_ at the second ventilatory threshold (9, 10). The V˙O_2max_ values of the male elite SkiMo athletes are >70 ml/min/kg (10). Unfortunately, to date, less comparison of these key performance components between male and female athletes has been published in SkiMo. Moreover, the definition of an “elite athlete” is often vague and subjective. In this article, we will use the terms “elite” and “world Class” athletes’, referring to tiers 4 and 5 of the classification proposed by McKay et al. (11).

In endurance sport, there is limited data available on elite or world class female runners, but typically, their V˙O_2max_ values are around 10%–15% lower than those observed in male runners when expressed in ml/min/kg (e.g., 67.1 vs. 74.1 ml/min/kg) (12), and even 20% lower if the highest V˙O_2max_ values ever reported so far in women and men are compared [80 (13) vs. 96 ml/min/kg (14), respectively]. In winter sports, recent data (4) on sex-differences in V˙O_2max_ expressed in relative value (ml/min/kg) has been recently published on cross-country ski (10%–27%), biathlon (18%–27%) and speed skating (12%–25%). The lower V˙O_2max_ value of elite female athletes compared to their male counterparts is linked to central factors, such as smaller heart and lung sizes, as well as lower hemoglobin mass, which limit their oxygen transport capacity and consequently the oxygen delivery to the working muscles (15, 16). Additionally, these differences in V˙O_2max_ when expressed in kg of body mass are also directly impacted by the higher body fat ratio in women (17). Potter et al. (18), reported that fit women typically have 8%–10% more fat mass than equally fit men (e.g., 24% vs. 16%). In SkiMo sprint events, an additional determinant of performance may be the anaerobic capacity. Measuring anaerobic energy production accurately and reliably is more challenging than assessing aerobic production, which is why there are only few studies on sex differences in anaerobic power (19). Anaerobic energy production during supramaximal exercise has been estimated across various sports using the accumulated oxygen deficit. In short, high-intensity activities lasting around 3 min, such as cross-country sprints, track cycling, and rowing, the oxygen deficit accounts for approximately 26%–27% of the total energy expenditure (20, 21). While oxygen deficit has not been measured specifically in SkiMo sprints, one can reasonably speculate that it would be similar to that previously reported in sports of similar duration, suggesting that anaerobic capacity is likely a key component of performance in Skimo sprint events. Males and females also differ in anaerobic power. With more contractile tissue, males’ larger muscle mass enables them to generate greater metabolic power anaerobically, giving them an advantage in this area (19).

Given that V˙O_2max_ and %BF are known as key factors in SkiMo performance, and that anaerobic power may also be important, one may anticipate significant differences between male and female performances. Moreover, a recent study (7) analyzed the time spent during different sections of two sprint races in the 2022 season. In the first race, the leading female completed the initial uphill section in 84.6 s, while the leading male completed it in 67.6 s, showing a 20.9% difference between them. The difference was slightly lower for the second sprint, which was less steep, with a 16.5% difference (88.2 s vs. 73.6 s). These differences align with data reported across six sports involving uphill performance (8), where the mean differences in performance between females and males were as follows: 34.1% in speed climbing, 22.8% in vertical SkiMo races, 20.0% in vertical kilometer running, 18.4% in uphill cross-country skiing races, 31.5% in an uphill section of road cycling, and 19.7% across three iconic uphill segments of the UTMB in trail running.

Sex comparison of physiological factor could help to explain these differences that seems to be higher in comparison to horizontal displacement like marathon road running (10%–12%) (22). Overall the above-reported sex-differences in uphill performance (18%–34%) (8) appear to be larger than those reported in relative V˙O_2max_ (10%–20%) at elite level (4, 19), when V˙O_2max_ is expressed in kg of body mass (and therefore takes into account the differences in fat mass). To the best of our knowledge, and as confirmed by a recent review (4), no data is currently available on sex differences in these physiological factors among elite SkiMo athletes. However, understanding the differences in aerobic capacity between elite/worldclass male and female SkiMo athletes would be valuable, particularly for coaches seeking to optimize training programs. Therefore, the aim of this study was to analyze sex differences in physiological parameters obtained during a graded exercise test to exhaustion at a slope of 25% on elite male and female SkiMo athletes. We tested the hypothesis that the sex difference in maximal vertical velocity would be larger than the sex difference in V˙O_2max_ among 20 elite SkiMo athletes.

## Methods

Twenty elite ski mountaineering athletes (6 females and 14 males) from the Swiss national team, classified as elite or world class level (11), participated in this study. They were aged 20–32 years old. All athletes had previously competed in several SkiMo events at the World Cup level. Of the 20 athletes, 13 have already achieved at least one podium finish in the World Cup. Six of them have secured at least one podium at the World Championships, and four have reached the podium at the European Championships. All athletes were informed about the procedures and provided written informed consent before the start of the experimental protocol. Approval for this study was obtained from the institutional ethical committee (CER-VD 2023-01638) and was in accordance with the Declaration of Helsinki.

### Protocol

All participants were instructed to avoid caffeine, alcohol, and intense exercise on the day prior to the tests. Upon arrival, classic anthropometric measurements were taken first (weight, height and body composition). Then they performed a walking/running incremental exercise to exhaustion performed at a slope of 25%.

Since a growing body of literature shows that exercise performance, including in hypoxia (23), is not different between the different phases of the menstrual cycle in eumenorrheic women (24), the present protocol did not schedule the female athletes at a specific phase of their menstrual cycle (but this information was recorded for on-going intra-athlete monitoring).

### Anthropometric assessment

Body mass was measured with a digital scale (Trisa, Switzerland) to the nearest 0.1 kg. Height was measured with a wall-mounted stadiometer to the nearest 0.1 cm. For twelve athletes (4 females; 8 males), a-posteriori measurement of the body composition was assessed using the skinfold method by a trained technician. Skinfold thickness was measured at six different sites (calf, medial thigh, suprailiac, abdominal, triceps, subscapular) with the Holtain Skinfold Caliper (Holtain Ltd, Crymych, UK). The caliper's dial provides measurements in 0.2 mm increments. Each measurement was taken three times, and the average was used to estimate the %BF of the athletes using Yuhasz's equation (25).

### Physiological assessment

The graded exercise test (GXT) to exhaustion was conducted on a motorized treadmill (Cosmed T170, Rome, Italy) set at a constant 25% slope. Trail running poles were used by the athletes throughout the test.

During the GXT, the treadmill slope remained constant, and the speed was increased each minute by 0.3 km/h to raise the intensity, corresponding to a 75 m/h increase in vertical velocity (vV). Athletes began the test walking and could freely transition to running. Females started the test at 3.5 km/h (875 m/h) and males at 4.0 km/h (1,000 m/h) They were secured with a harness and instructed to continue until exhaustion. A recovery period of 20 min was then assessed while the participants remained seated on a chair.

Gaz exchange measurements were continuously collected using a breath-by-breath analyzer (Quark CPET, Cosmed, Rome, Italy). Flow sensors and gas analyzers were carefully calibrated before each measurement according to the manufacturer's instructions. Heart rate (HR) signals were recorded using a commercially available device, the Polar H10 HR sensor (Polar, Finland). Pulse oxygen saturation (SpO_2_) was continuously recorded at the earlobe using the Nonin 8000Q2 earclip (Nonin Medical Inc., Plymouth, MN) integrated into the gas analyzer. Lactate measurements were taken throughout the recovery period after 1, 3, 6, 9, 12, 16, and 20 min. A 20 μl blood sample from the finger was collected using Unistik 3 Extra Lancets (Owen Mumford, Woodstock, UK) and drawn with a sterile glass capillary tube (Sanguis Counting, Germany). The sample was then mixed with 1 ml of haemolyzing solution. Samples were gently inverted to mix thoroughly before lactate concentration was analyzed using the Biosen C-line Clinic measurement system (EKF Diagnostics, Cardiff, UK).

### Data analysis

Gas exchange data were analyzed using MATLAB (Version R2023b; The MathWorks, Inc., Natick, MA, USA). V˙O₂_max_ was defined as the highest 20s-average value. First and second ventilatory thresholds (VT_1_, VT_2_) were determined by visual inspection, as described by Beaver et al. (26). The average of the 20 s preceding both thresholds was calculated for all parameters. Regarding vV (m/h), the velocity of the last completed level was recorded for each athlete. The vertical running economy (REv, ml/kg/vertical km) which refers to the oxygen cost at a given running speed was calculated by taking the average V˙O_2_ values at VT_1_, VT_2_, and MAX, respectively divided by the corresponding vV at each intensity level. The maximal blood lactate concentration value ([BLa]_max_) was defined as the highest measurement recorded during the recovery period.

### Statistical analyses

Data is reported as mean ± SD. The Shapiro–Wilk test was employed to assess normal distribution. To compare mean values between performance groups, either an independent *t*-test or the Mann–Whitney *U* test was applied, depending on the distribution of the data. Statistical significance was set at *p* < 0.05. Effect size was calculated using Cohen's *d* coefficient for Student's *t*-tests. For the Mann–Whitney test, effect size was represented by rank biserial correlation. Relationship between V˙O_2max_ and vV was assessed using Pearson's correlation coefficient. Statistical analyses were undertaken using SPSS version 20.0 (SPSS Inc., Chicago, IL, USA), and all figures were created on Prism 8 (Graphpad Software INC., La Jolla, CA, USA).

## Results

### General characteristics

Sex differences between male and female athletes for general characteristics, are presented in Table 1. As expected, the height and weight of males were significantly greater than those of females. Females had a significantly higher %BF, although there was no significant difference in BMI between males and females.

**Table 1:** Athletes’ main characteristics.

		Females	Males	Sex effect	ES
Characteristics	Subjects (n)	6	14	n/a	n/a
	Age (years)	25 ± 3	24 ± 4	0.554	0.5
	Height (cm)	169 ± 6	182 ± 5	**<0**.**001**	0.8
	Weight (kg)	60.1 ± 6.2	70.9 ± 7.3	**0**.**006**	0.6
	BMI (kg m^−2^)	21.0 ± 1.2	21.3 ± 1.3	0.594	0.5
	%BF (%)	15.2 ± 1.0	6.6 ± 0.6	**0**.**004**	0.3$

BMI, body mass index; %BF, percent body fat (*n* = 4 females and 8 males). *P* < 0.05 for statistical differences between males and females. Values in bold: *P* < 0.05 for statistical differences between males and females. Effect size (ES) is reported as Cohen's *d* when applicable or as the rank biserial correlation ($) when the Mann–Whitney test is used.

### GXT

**Figure 1:**
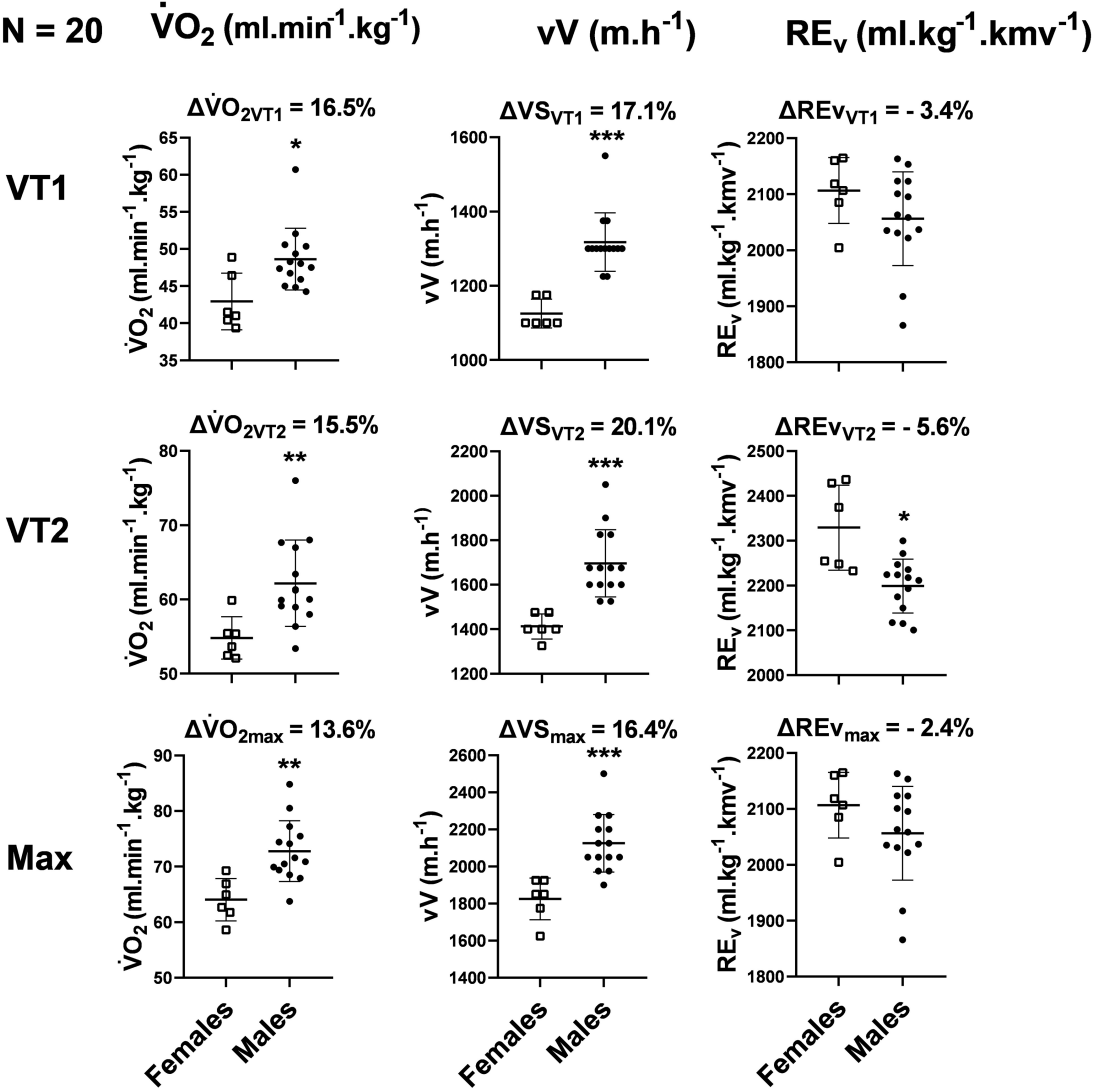
Percent differences between females and males, using the mean value for females as reference. V˙O_2_: Oxygen consumption. vV, vertical velocity; Rev, vertical running economy. All three parameters are represented at the first ventilatory threshold (VT_1_), the second ventilatory threshold (VT_2_), and at maximum (Max). *p < 0.05 for differences between females and males.

Sex differences between males and females during the GXT are presented in Table 2. Differences in percent between females and males are shown in Figure 1. Elite female SkiMo athletes had a V˙O_2_ value 13.6% lower at MAX and 15.5% lower at VT_2_ than their male counterparts. Interestingly, the sex-differences in vV at both MAX and VT_2_ intensities were consistently larger than the differences in V˙O_2_. Maximal blood lactate concentration tended to be lower for females; however, the difference was not statistically significant (*p* = 0.066). Maximal blood lactate concentration appeared after 1 min for 15 athletes and after 3 min for the five others.

**Table 2:** Athletes’ cardiopulmonary parameters at the first (VT_1_) and second (VT_2_) ventilatory threshold and maximal values (Max).

		Females	Males	Sex effect	ES
VT_1_	SpO_2_ (%)	98.4 ± 0.9	98.2 ± 1.0	0.646	0.5
	V˙_E_ (L min^−1^)	62.1 ± 3.4	82.9 ± 11.2	**<0**.**001**	0.6
	HR (beats min^−1^)	156 ± 7	154 ± 8	0.631	0.5
	V˙O_2_ (ml min^−1^ kg^−1^)	42.9 ± 3.8	48.6 ± 4.2	**0**.**017**	0.3$
	V˙O_2_ (%)	67.6 ± 5.0	68.0 ± 2.5	0.861	0.5
	vV (m h^−1^)	1,125 ± 39	1,318 ± 79	**<0**.**001**	0.3$
	RE_v_ (ml kg^−1^ kmv^−1^)	2,291 ± 211	2,213 ± 98	0.264	0.5
VT_2_	SpO2 (%)	98.0 ± 0.8	96.9 ± 1.8	0.328	0.3$
	V˙_E_ (L min^−1^)	89.6 ± 10.8	126.8 ± 14.5	**<0**.**001**	0.9
	HR (beats min^−1^)	175 ± 8	179 ± 7	0.260	0.5
	V˙O_2_ (ml min^−1^ kg^−1^)	54.8 ± 2.8	62.2 ± 5.8	**0**.**009**	0.6
	V˙O_2_ (%)	86.2 ± 4.5	86.9 ± 3.7	0.717	0.5
	vV (m h^−1^)	1,412 ± 56	1,696 ± 151	**<0**.**001**	0.8
	RE_v_ (ml kg^−1^ kmv^−1^)	2,329 ± 95	2,199 ± 60	**0**.**018**	0.7
Max	SpO_2_ (%)	92.5 ± 3.7	94.0 ± 2.7	0.580	0.6
	V˙E (L min^−1^)	126.7 ± 8.4	187.4 ± 13.7	**<0**.**001**	1.5
	HR (beats min^−1^)	186.7 ± 8.7	191.4 ± 5.1	0.261	0.5
	V˙O_2_ (ml min^−1^ kg^−1^)	64.0 ± 3.8	72.8 ± 5.5	**0**.**002**	0.7
	vV (m h^−1^)	1,825 ± 113	2,125 ± 156	**<0**.**001**	0.7
	REv (ml kg^−1^ kmv^−1^)	2,107 ± 59	2,056 ± 84	0.202	0.5
	[BLa]_max_ (mmol L^−1^)	10.6 ± 1.6	12.5 ± 2.1	0.066	0.6

SpO_2_, pulse oxygen saturation; V˙E, minute ventilation; HR, heart rate; V˙O_2_, oxygen uptake; vV, vertical velocity; REv, running vertical economy; [BLa]_peak_, peak blood lactate concentration.

Values in bold: *P* < 0.05 for statistical differences between males and females. Effect size (ES) is reported as Cohen's *d* when applicable or as the rank biserial correlation ($) when the Mann–Whitney test is used.

## Discussion

To our knowledge, this is the first study reporting sex differences in aerobic capacity and performance metrics in elite-level ski mountaineers. The main findings of this study are: (i) Sex differences in vertical velocity (16.4–20.1%) exceed those in V˙O_2_ (13.6–16.5%) when comparing elite/world-class female and male SkiMo athletes at VT_1_, VT_2_, and MAX. (ii); Furthermore, %BF was higher in females, despite similar BMI values between males and females. (iii); Finally, vertical running economy at VT_2_ was significantly less efficient in females compared to males.

**Figure 2:**
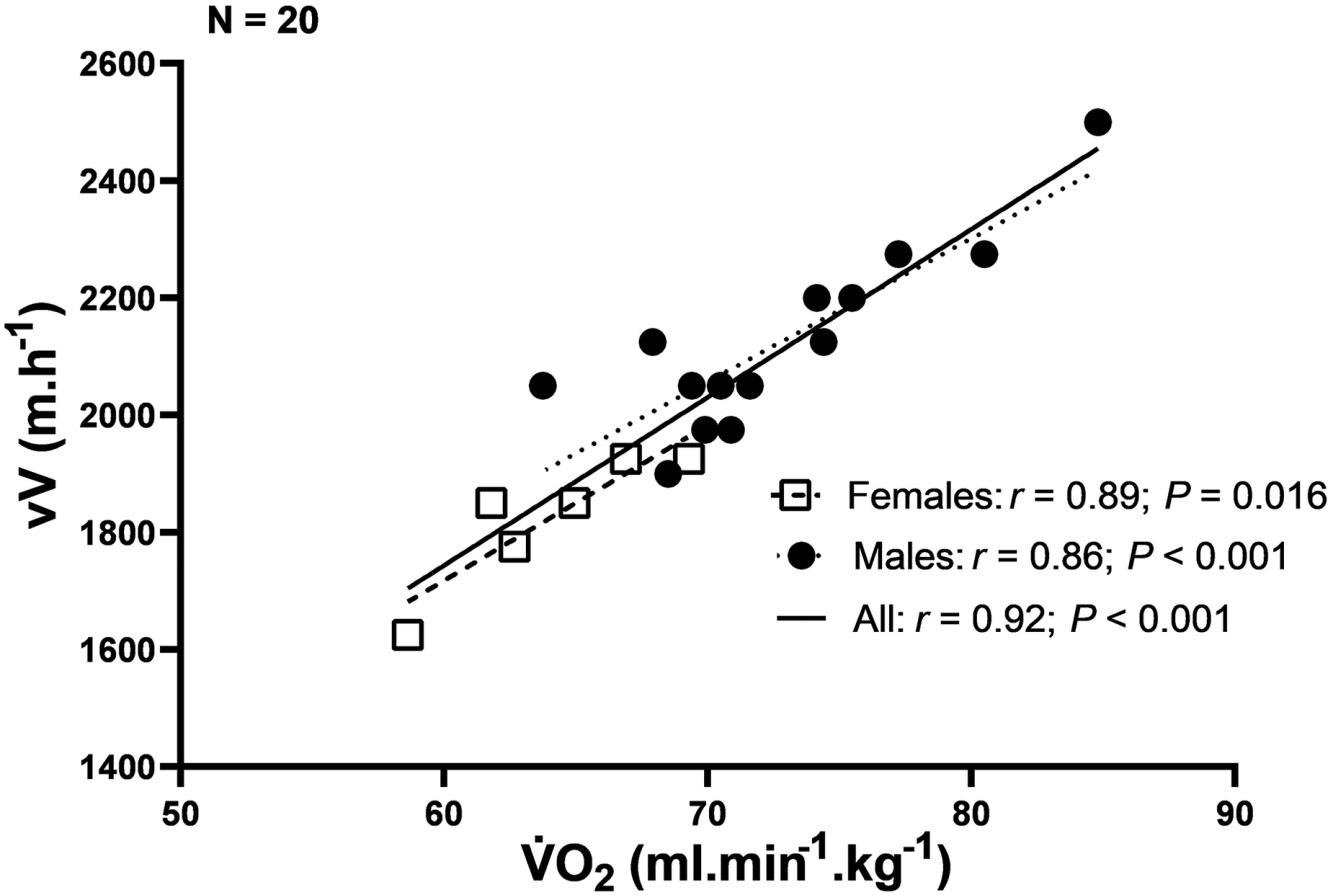
Correlation between vertical velocity (vV) and maximal oxygen consumption (V˙O_2max_).

Aerobic power is the most important parameter of aerobic endurance and the main factor underlying the sex differences observed in endurance performance (19). Our results confirm the strong relationship between V˙O_2max_ and vV, as shown in Figure 2. Moreover, aerobic power in males align with previous studies [i.e., V˙O_2max_ > 70 ml/kg/min; VT_1_ and VT_2_ at ∼70% and 90% (5, 9, 10, 27)] but is complemented by the present data on elite female SkiMo athletes, who exhibit V˙O_2max_ values around 64 ml/kg/min, with similar percentages for the ventilatory thresholds as their male counterparts. As illustrated in Figure 1, the sex difference in V˙O_2max_ (13.6%) aligns with the known 10%–15% difference commonly reported in elite athletes runners (17). When compared to other prominent winter sports, such as cross-country skiing, Tønnessen et al. (28), reported V˙O_2max_ values for Olympic medalists and non-medalists in Nordic skiing, with mean ranges of 62–73 ml/min/kg for women and 73–84 ml/min/kg for men, slightly higher than those reported in the present study. This difference could suggest a greater competitive density in cross-country skiing, a sport long established in the winter Olympics, possibly explaining the higher V˙O_2max_ values observed in these athletes. Maximal aerobic power is known as a key factor in SkiMo performance, but analyzing vertical velocity is particularly valuable and practical since it can be easily measured on field with GPS for understanding performance outcomes. In the present study, the differences between men and women were consistently more pronounced in terms of vV than in V˙O_2_ (Figure 1), which leads us to accept our main hypothesis. Females had a lower V˙O_2_ value than men at all three intensities analyzed, and the difference in vV was greater each time. When comparing speed at each intensity analyzed, we also observed a larger difference than what is commonly reported (10%–12%) in elite marathon runners (22). Our results, showing (16.4–20.1%) difference between females and males, are more in line with those of Millet & Malatesta (29), who reported a mean difference of 16%–17% in time performance across three ultra-trail marathons and with a recent article (8) where a 18.4% sex difference in uphill performance vs. 9.4% on flat terrain was reported over 10 years at world-level in cross-country skiing. Similarly, the analysis of 10 years of “vertical race” event in the World cup showed that females were on average 22.8% slower than males (8). Additionally, our findings on sex differences align with previous ones (4), particularly with those of Solli and Losnegard (30), who reported a 17%–24% difference between females and males during a 3-min uphill test conducted in a cross-country skiing laboratory setting. These recent studies, along with our findings, underscore the larger performance gap between males and females in uphill sport locomotion. Further investigation is needed to understand the underlying mechanisms, but several factors may contribute to this pronounced sex difference in uphill velocity beyond aerobic power alone (19).

### Body composition

Since SkiMo performance has been shown to have a strong correlation with V˙O_2max_ when expressed as relative values (ml/kg/min), optimizing an athlete's body mass has been suggested as an important factor (5, 27). Additionally, a recent study by Fornasiero (9) demonstrated that fat mass (kg) and %BF were negatively correlated with uphill SkiMo performance. However, no correlation was found between body mass (kg) and performance. This suggests that the favorable anthropometric traits of top ski mountaineers are related not only to body weight but also to body composition (9). Therefore, assessing body composition, alongside body mass, appears essential for accurately evaluating elite ski mountaineers. A recent review (31), reported that %BF was negatively associated with performance in prolonged endurance events. Some studies (32, 33) suggested that increasing lean body mass, particularly in the upper body, while reducing fat mass may be the most favorable strategy for improving performance, particularly in female Nordic skiing athletes.

Our results demonstrate that BMI is not significantly different between females and males, however, there remained a significant difference in V˙O_2max_ values, confirming that sex differences in maximal aerobic capacity are not solely influenced by BMI (34). This discrepancy can be partially attributed by the difference in %BF between males and females. As expected, in our study, %BF was significantly higher in females compared to males, with a mean difference of 8.6%, aligning with the findings of Potter et al. (18). Additionally, the present %BF values for elite female athletes (15.2%) are comparable to those reported for elite female runners (14.3%) (12). Given that V˙O_2max_ and %BF are strongly correlated with uphill performance (9, 35), it is consistent that the male-female disparity in vertical velocity is more pronounced on test performed at a 25% slope. Females appear to be more disadvantaged compared to males when running/walking uphill, as opposed to horizontal movements. However, it is important to note that there is a high risk that suggesting body fat standards that are biologically unrealistic—by being overly stringent—may compromise physical and mental health by encouraging unhealthy weight management behaviors or favoring individuals with suboptimal performance (36). This approach can also increase the risk of relative energy deficiency in sport (REDs), further impairing both performance and overall well-being (37). The aim here is not to suggest that females should reduce their body fat ratio at all costs, but rather to highlight a greater performance difference when moving uphill, as females naturally require more adipose tissue than males to maintain good health.

Several additional parameters may explain the performance gap between males and females in uphill locomotion, though they were not examined in our study. We will briefly outline these factors here. Firstly, females typically have a greater proportion of slow-twitch oxidative fibers and a lower proportion of fast-twitch fibers (38). These differences may place them at a disadvantage during climbing, where performance is heavily reliant on concentric contractions and high force production. Additionally, males tend to have a greater muscle cross-sectional area and higher upper body muscle mass than females (39, 40), which may account for the higher absolute power output observed in males. Lastly, the pulmonary system reveals marked sex-related differences under hypoxic conditions, with females showing greater susceptibility to hypoxemia and an increased work of breathing compared to men (41). Together, these physiological differences may contribute to the observed performance gap in mountain contexts.

### Vertical running economy

Running economy (RE) is recognized as one of the most reliable predictors of running performance among homogeneous groups of trained athletes (42). How important RE is for mountain/trail running is still a subject of debate (43). However, it seems to be a key component of uphill locomotion since RE on level vs. uphill running was correlated in elite mountain runners (44). Moreover, gross efficiency (GE) was highly correlated with vertical SkiMo performance (9). While skiing or running efficiency is challenging to measure directly from an engineering perspective, RE—which refers to the oxygen cost at a given running speed—is often used as an indicator of efficiency (17). It is known that there is no clear difference between males and females in this parameter (45). In the present study, REv in males was consistently slightly better, particularly when considering oxygen consumption relative to body weight and per vertical kilometer. However, the sex difference was statistically significant only at the second ventilatory threshold, indicating greater efficiency in males at this intensity. At maximal effort, since males tended to have a higher peak lactate (12.5 ± 2.1 vs. 10.6 ± 1.6, *p* = 0.066), one may speculate that their anaerobic component was somewhat higher, enabling them to reach a slightly higher vV. This would increase the V̇O_2_/speed ratio in females compared to males, contributing that REv was not significantly different between males and females. However, our data on [BLa]_max_ aligns with other studies where [BLa]_max_ is generally quite similar between males and females (46–48). One might expect females to have lower [BLa]_max_ due to their lower muscle mass. However, factors such as a higher percentage of adipose tissue, a lower blood volume, and potentially elevated blood epinephrine levels could offset the difference in total lactate production, resulting in peak blood lactate concentrations comparable to those in males (47).

### Strengths and limitations

The main strength of this study relies on it novelty since (i) there is very little data on elite and world-class SkiMo athletes and (ii) their physiological characteristics cannot be inferred from subelite data (49). However, this study has slight limitations: body composition measurement was only performed on 12 out of 20 participants, due to logistical and time constraints. Despite this, we believe the data from these 4 females and 8 males accurately reflect the characteristics of the two groups. Second, the gender distribution was unbalanced, with 14 males and 6 females, which may have introduced potential issues related to sample size. One other potential limitation of this study relates to the graded exercise test protocol, which inherently involves transitions in gait (e.g., from walking to running) as speed increases. While this mirrors the progressive nature of SkiMo competition, such transitions may introduce variability in gait efficiency, potentially influencing data collection and interpretation. Walking and running represent distinct exercise modalities, and the efficiency differences between these gaits could impact the observed physiological responses, particularly at higher intensities. This raises an interesting question about the interplay between gait transition and performance, especially regarding the sex differences observed at VT2 but not at VT1. Future studies could explore protocols starting at a running pace or employing a speed-elevation function to better assess efficiency across transitions.

## Conclusion

The main results of this study are: (i) The sex-differences in vertical velocity are larger than those in V˙O_2_ when comparing elite SkiMo female and male athletes at VT_1_, VT_2_, and at maximal effort. (ii); Percent body fat is higher for females. (iii); The vertical running economy is significantly less efficient in females compared to males at VT_2_.

Further investigation on anaerobic capacity and on muscle composition in upper and lower limbs would help to further elucidate additional mechanisms.

## Data Availability

The raw data supporting the conclusions of this article will be made available by the authors, without undue reservation.

## References

[1] Schöffl VR Zimmermann P Küpper T Lutter C. Ski mountaineering—scientific knowledge of this new Olympic sport: a narrative review. Curr Sports Med Rep. (2023) 22(2):61–6. 10.1249/JSR.0000000000001038 36757125

[2] Bortolan L Savoldelli A Pellegrini B Modena R Sacchi M Holmberg HC Ski mountaineering: perspectives on a novel sport to be introduced at the 2026 winter Olympic games. Front Physiol. (2021) 12:737249. 10.3389/fphys.2021.737249 34744777 PMC8566874

[3] Schöffl VR Bösl T Lutter C. Ski mountaineering: sports medical considerations for this new Olympic sport. Br J Sports Med. (2022) 56(1):2–3. 10.1136/bjsports-2021-104846 34836881

[4] Solli GS Sandbakk Ø McGawley K. Sex differences in performance and performance-determining factors in the Olympic winter endurance sports. Sports Med-Open. (2024) 10(1):126. 10.1186/s40798-024-00792-8 39565528 PMC11579258

[5] Duc S Cassirame J Durand F. Physiology of ski mountaineering racing. Int J Sports Med. (2011) 32(11):856–63. 10.1055/s-0031-1279721 22012642

[6] Fornasiero A Callovini A Fornoni S Savoldelli A Schena F Holmberg HC Participation and performance by women and men in ski-mountaineering sprint races during the past decade. J Sports Med Phys Fitness. (2023) 63(6):707–12. 10.23736/S0022-4707.23 36790327

[7] Fornasiero A Fornoni S Callovini A Todesco B Savoldelli A Schena F Analysis of sprint ski mountaineering performance. Int J Sports Physiol Perform. (2023) 19(2):155–63. 10.1123/ijspp.2023-0075 38086366

[8] Millet GP Raberin A Faiss R Giovanelli N Galindo T Place N Women upward—sex differences in uphill performance in speed climbing, ski mountaineering, trail running, cross-country skiing, and cycling. Int J Sports Physiol Perform. (2024) 20(2):246–55. 10.1123/ijspp.2024-0354 39732139

[9] Fornasiero A Savoldelli A Boccia G Zignoli A Bortolan L Schena F Physiological factors associated with ski-mountaineering vertical race performance. Sport Sci Health. (2018) 14:97–104. 10.1007/s11332-017-0407-0

[10] Lasshofer M Seifert J Wörndle AM Stöggl T. Physiological responses and predictors of performance in a simulated competitive ski mountaineering race. J Sports Sci Med. (2021) 20(2):250–7. 10.52082/jssm.2021.250 34211317 PMC8219267

[11] McKay AKA Stellingwerff T Smith ES Martin DT Mujika I Goosey-Tolfrey VL Defining training and performance caliber: a participant classification framework. Int J Sports Physiol Perform. (2021) 17(2):317–31. 10.1123/ijspp.2021-0451 34965513

[12] Pate RR O’Neill JR. American women in the marathon. Sports Med. (2007) 37(4–5):294–8. 10.2165/00007256-200737040-00006 17465592

[13] Haugen T Paulsen G Seiler S Sandbakk O. New records in human power. Int J Sports Physiol Perform. (2017) 13(6):678–86. 10.1123/ijspp.2017-0441 28872385

[14] Rønnestad BR Hansen J Stensløkken L Joyner MJ Lundby C. Case studies in physiology: temporal changes in determinants of aerobic performance in individual going from alpine skier to world junior champion time trial cyclist. J Appl Physiol. (2019) 127(2):306–11. 10.1152/japplphysiol.00798.2018 31194601

[15] Santisteban K Lovering A Halliwill J Minson C. Sex differences in VO_2max_ and the impact on endurance-exercise performance. Int J Environ Res Public Health. (2022) 19(9):4946. 10.3390/ijerph19094946 35564339 PMC9105160

[16] Millet GP Burtscher J Bourdillon N Manferdelli G Burtscher M Sandbakk Ø. The V˙O_2max_ legacy of hill and lupton (1923)—100 years on. Int J Sports Physiol Perform. (2023) 18(11):1362–5. 10.1123/ijspp.2023-0229 37770066

[17] Joyner MJ. Physiological limits to endurance exercise performance: influence of sex. J Physiol. (2017) 595(9):2949–54. 10.1113/JP272268 28028816 PMC5407964

[18] Potter AW Tharion WJ Nindl LJ McEttrick DM Looney DP Friedl KE. The normal relationship between fat and lean mass for mature (21–30 year old) physically fit men and women. Am J Hum Biol. (2024) 36(1):e23984. 10.1002/ajhb.23984 37695262

[19] Sandbakk Ø Solli GS Holmberg HC. Sex differences in world-record performance: the influence of sport discipline and competition duration. Int J Sports Physiol Perform. (2018) 13(1):2–8. 10.1123/ijspp.2017-0196 28488921

[20] Losnegard T Myklebust H Hallén J. Anaerobic capacity as a determinant of performance in sprint skiing. Med Sci Sports Exerc. (2012) 44(4):673–81. 10.1249/MSS.0b013e3182388684 21952633

[21] Gastin PB. Energy system interaction and relative contribution during maximal exercise. Sports Med. (2001) 31(10):725–41. 10.2165/00007256-200131100-00003 11547894

[22] Hunter SK Stevens AA Magennis K Skelton KW Fauth M. Is there a sex difference in the age of elite marathon runners? Med Sci Sports Exerc. (2011) 43(4):656–64. 10.1249/MSS.0b013e3181fb4e00 20881885

[23] Citherlet T Raberin A Manferdelli G Pialoux V Millet GP. Menstrual cycle does not impact the hypoxic ventilatory response and acute mountain sickness prediction. Sci Rep. (2024) 14(1):26087. 10.1038/s41598-024-76404-y 39477965 PMC11525676

[24] Elliott-Sale KJ McNulty KL Ansdell P Goodall S Hicks KM Thomas K The effects of oral contraceptives on exercise performance in women: a systematic review and meta-analysis. Sports Med. (2020) 50(10):1785–812. 10.1007/s40279-020-01317-5 32666247 PMC7497464

[25] Yuhasz MS. Physical Fitness Manual. London, ON: University of Western Ontario (1977). p. 204.

[26] Beaver WL Wasserman K Whipp BJ. A new method for detecting anaerobic threshold by gas exchange. J Appl Physiol. (1986) 60(6):2020–7. 10.1152/jappl.1986.60.6.2020 3087938

[27] Schenk K Faulhaber M Gatterer H Burtscher M Ferrari M. Ski mountaineering competition: fit for it? Clin J Sport Med. (2011) 21(2):114–8. 10.1097/JSM.0b013e31820f903e 21358501

[28] Tonnessen E Haugen TA Hem E Leirstein S Seiler S. Maximal aerobic capacity in the winter-olympics endurance disciplines: olympic-medal benchmarks for the time period 1990–2013. Int J Sports Physiol Perform. (2015) 10(7):835–9. 10.1123/ijspp.2014-0431 25611016

[29] Millet GP Malatesta D. Sex differences in human running performance: what about mountain ultramarathon? J Appl Physiol. (2022) 133(6):1300–1. 10.1152/japplphysiol.00506.2022 36455549

[30] Sollie O Losnegard T. Sex differences in physiological determinants of performance in elite adolescent, junior, and senior cross-country skiers. Int J Sports Physiol Perform. (2022) 17(8):1304–11. 10.1123/ijspp.2021-0366 35894954

[31] Mathisen T Ackland T Burke L Constantini N Haudum J Macnaughton L Best practice recommendations for body composition considerations in sport to reduce health and performance risks: a critical review, original survey and expert opinion by a subgroup of the IOC consensus on relative energy deficiency in sport (REDs). Br J Sports Med. (2023) 57(17):1148–58. 10.1136/bjsports-2023-106812 37752006

[32] Jones T Lindblom H Karlsson Ø Andersson E McGawley K. Anthropometric, physiological, and performance developments in cross-country skiers. Med Sci Sports Exerc. (2021) 53(12):2553–64. 10.1249/MSS.0000000000002739 34649265

[33] Jones NL Jones GL Obminski G Satia I Killian KJ. Maximum exercise capacity: barcroft revisited in a large population. Can J Respir Crit Care Sleep Med. (2024) 8(6):260–9. 10.1080/24745332.2024.2397052

[34] Helgerud J. Maximal oxygen uptake, anaerobic threshold and running economy in women and men with similar performances level in marathons. Eur J Appl Physiol. (1994) 68(2):155–61. 10.1007/BF00244029 8194545

[35] Alvero-Cruz JR Parent Mathias V Garcia Romero J Carrillo de Albornoz-Gil M Benítez-Porres J Ordoñez FJ Prediction of performance in a short trail running race: the role of body composition. Front Physiol. (2019) 10:1306. 10.3389/fphys.2019.01306 31681014 PMC6805720

[36] Foulis SA Hughes JM Walker LA Guerriere KI Taylor KM Proctor SP Body mass does not reflect the body composition changes in response to similar physical training in young women and men. Int J Obes. (2005) 45(3):659–65. 10.1038/s41366-020-00730-0 33414487

[37] Mountjoy M Ackerman KE Bailey DM Burke LM Constantini N Hackney AC 2023 International Olympic committee’s (IOC) consensus statement on relative energy deficiency in sport (REDs). Br J Sports Med. (2023) 57(17):1073–97. 10.1136/bjsports-2023-106994 37752011

[38] Hunter SK Butler JE Todd G Gandevia SC Taylor JL. Supraspinal fatigue does not explain the sex difference in muscle fatigue of maximal contractions. J Appl Physiol. (2006) 101(4):1036–44. 10.1152/japplphysiol.00103.2006 16728525

[39] Hegge AM Bucher E Ettema G Faude O Holmberg HC Sandbakk Ø. Gender differences in power production, energetic capacity and efficiency of elite cross-country skiers during whole-body, upper-body, and arm poling. Eur J Appl Physiol. (2016) 116(2):291–300. 10.1007/s00421-015-3281-y 26476546

[40] Staron RS Hagerman FC Hikida RS Murray TF Hostler DP Crill MT Fiber type composition of the Vastus Lateralis muscle of young men and women. J Histochem Cytochem. (2000) 48(5):623–9. 10.1177/002215540004800506 10769046

[41] Raberin A Burtscher J Citherlet T Manferdelli G Krumm B Bourdillon N Women at altitude: sex-related physiological responses to exercise in hypoxia. Sports Med. (2024) 54(2):271–87. 10.1007/s40279-023-01954-6 37902936 PMC10933174

[42] McLaughlin J Howley E Bassett D Thompson D Fitzhugh E. Test of the classic model for predicting endurance running performance. Med Sci Sports Exerc. (2009) 42(5):991–7. 10.1249/MSS.0b013e3181c0669d 19997010

[43] Millet GP. Economy is not sacrificed in ultramarathon runners. J Appl Physiol. (2012) 113(4):686; author reply 687. 10.1152/japplphysiol.00642.2012 22896682

[44] Willis SJ Gellaerts J Mariani B Basset P Borrani F Millet GP. Level versus uphill economy and mechanical responses in elite ultratrail runners. Int J Sports Physiol Perform. (2019) 14(7):1001–5. 10.1123/ijspp.2018-0365 30676150

[45] Fletcher JR MacIntosh BR. Achilles tendon strain energy in distance running: consider the muscle energy cost. J Appl Physiol. (2015) 118(2):193–9. 10.1152/japplphysiol.00732.2014 25593218 PMC4297774

[46] Lehmann M Berg A Keul J. Sex-related differences in free plasma catecholamines in individuals of similar performance ability during graded ergometric exercise. Eur J Appl Physiol. (1986) 55(1):54–8. 10.1007/BF00422893 3698988

[47] Zhang JQ Ji L. Gender differences in peak blood lactate concentration and lactate removal. Ann Sports Med Res. (2016) 3(7):1088. Available at: https://www.jscimedcentral.com/jounal-article-info/Annals-of-Sports-Medicine-and-Research/Gender-Differences-in-Peak-Blood-Lactate-Concentration-and-Lactate-Removal-4515#

[48] Froberg K Pedersen PK. Sex differences in endurance capacity and metabolic response to prolonged, heavy exercise. Eur J Appl Physiol. (1984) 52(4):446–50. 10.1007/BF00943378 6540674

[49] Burke LM Whitfield J Hawley JA. The race within a race: starting together, finishing apart. Free Radic Biol Med. (2024) 227:367–78. 10.1016/j.freeradbiomed.2024.10.277 39395564

